# Digital Health Information Provided by Public Health Stakeholders on Colorectal Cancer Screening: A Systematic Evaluation

**DOI:** 10.3390/ijerph192315624

**Published:** 2022-11-24

**Authors:** Claudia Hasenpusch, Henriette Faßhauer, Annemarie Minow, Lena Kannengießer, Ilona Hrudey, Svenja Walter, Christoph Stallmann, Enno Swart, Stefanie March

**Affiliations:** 1 Department of Social Work, Health and Media, Magdeburg-Stendal University of Applied Sciences, 39114 Magdeburg, Germany; 2 Department of Studies and Teaching Medicine, University of Lübeck, 23567 Lübeck, Germany; 3 Institute of Social Medicine and Health Systems Research, Medical Faculty, Otto von Guericke University Magdeburg, 39120 Magdeburg, Germany

**Keywords:** colorectal cancer, health information, systematic evaluation, screening, Internet, cancer prevention

## Abstract

In the federal state of Germany, Saxony-Anhalt, colorectal cancer is the second most frequent cause of death among cancer patients. In order to identify cancer precursors early, colorectal cancer screenings are essential. In this context, health information contributes to informing individuals and imparting them with necessary knowledge to make a decision about (non-)utilization of preventive services. Numerous public health stakeholders (e.g., statutory health insurances) provide health information. This study aimed to evaluate the quality of web-based health information offered by public health stakeholders in Saxony-Anhalt, Germany. A systematic evaluation was used. A search was performed using pre-defined eligibility criteria and search terms. Two independent reviewers assessed the search results based on seven main categories (60 items) developed by the study team in line with the “Guideline Evidence-based Health Information”. In total, 37 materials from 16 different stakeholders were included and yielded a “mediocre quality” (median = 69%). The materials had only partially fulfilled the requirements of national recommendations for evidence-based health information. Access to digital health information regarding colon cancer screening was unsatisfactory, especially for individuals with auditory or visual impairments, due to use of inappropriate communication technologies. Further efforts are required to improve digital health information about colorectal cancer screening.

## 1. Introduction

Cancers of the intestinal tract, especially of the colon and the rectum, are among the three most common cancer diagnoses for both men and women in Germany [1]. For 2018, the Centre for Cancer Registry Data of the Robert Koch Institute registered 33,920 newly diagnosed cases among men and 26,710 new cases among women [2,3]. The lifetime prevalence for men is 5.9% and for women 5.0%. The risk of developing colorectal cancer increases with age. The median age of more than half of those first diagnosed with the disease is over 70 years (men: 72 years, women: 76 years) [2]. In this context, cancer screening is particularly important. Conducting cancer screenings has the potential to lower the incidence, morbidity and mortality rate and thus reduce strain for the healthcare sector on the one hand [4]. On the other hand, at the individual level, cancer screening can detect early stages of cancer and prevent further development, thus improving the quality of life of those affected [4]. In the federal state of Germany, Saxony-Anhalt (2.2 million inhabitants) [5], the second most frequent cause of cancer-related deaths is malignant cancer of the intestine (deaths in 2019: 564 men, 427 women) [6]. A national statistical comparison related to colorectal cancer reveals that men in Saxony-Anhalt have the highest age-standardized incidence and fatality rates [3], with the number of excess deaths among men (58 cases) being above the national average [7]. 

In order to identify cancer precursors early, health-insured inhabitants in Germany have the opportunity to make use of the population-based, organized colorectal cancer screening program [1]. In 2018, the Federal Joint Committee (Gemeinsamer Bundesausschuss: G-BA), as the highest body of the joint self-government of service providers and health insurers in Germany, adopted the Guideline for Organized Cancer Screening Programs (oKFE-RL) [8]. It regulates, inter alia, a uniform procedure for the dissemination of information and introduced obligatory uniform informational materials produced by the G-BA. Since the adoption of the guideline, the Statutory Health Insurance (SHI) have sent all eligible insured persons aged 50 and older a personal invitation from the Federal Joint Committee to participate in the cancer screening, along with further informational materials from the G-BA about participation in the examination [8,9,10]. In Germany, people aged between 50 and 54 are entitled to an annual fecal occult blood test (FOBT). In addition, men aged 50 and over and women aged 55 and over can have colonoscopies performed at ten-year intervals, or, alternatively, from 55 years of age they are eligible for an FOBT every two years if they do not utilize the opportunity to have a colonoscopy [11]. These services are offered and carried out by, inter alia, service providers in outpatient health care (e.g., general practitioners). The costs for cancer screenings are covered by the SHI. Participation is voluntary [8]. 

The results of a longitudinal study (from 2009 to 2018) based on nationwide claims data of SHI-accredited physicians demonstrated a continuous decrease in the uptake of the FOBT and of the colonoscopy in higher age groups. In Saxony-Anhalt, the utilization rate for the FOBT in 2018 among men was below the national rate (Saxony-Anhalt: 6.5% vs. national rate: 7.4%) and among women it was above (Saxony-Anhalt: 29.0% vs. national rate: 23.4%) [12]. Further, 2.5% of men and 2.7% of women in Germany aged between 55 and 64 years old utilized the preventive colonoscopy. The utilization rate for the colonoscopy in Saxony-Anhalt was below the national rate of 2.6% for both men (2.0%) and women (2.2%) [13]. Due to the comparatively high incidence and mortality rates and the low uptake of colorectal cancer screening in Saxony-Anhalt, it seems important to provide targeted information about screenings. 

Health information plays an essential role in the uptake of preventive services because it makes a significant contribution towards enabling individuals to make informed and participatory health-related decisions [14,15,16,17]. According to the “Good Practice Health Information“ of the German Network for Evidence-based Medicine [18] as well as other sources, health-related information should be neutral, balanced, in line with the current state of evidence-based medicine, comprehensible and transparent, with the goal of providing reliable information about the benefits and risks [14,18,19,20]. At the health policy level there is an increasing focus on demanding quality-assured and patient-oriented health information. Firstly, in 2013, on the basis of the National Cancer Plan, a new law was adopted that provided a legal basis for the further conceptual development of cancer screening [4,21]. One of the main goals was to improve the information for insured persons, especially with regard to the clarification of the benefits and risks of screening procedures [21]. Secondly, the Patients’ Rights Act required information provided to patients to be understandable for the patient (German Civil Code §630e, subsection 2, sentence 3) [22]. 

Numerous public health stakeholders at a federal and state level provide information on colorectal cancer screening in various media forms (e.g., flyers, posters, websites) [19]. Of these, Internet-based health information constitutes an increasingly important and accessible source of information on health topics [23]. Compared to analog media, the Internet enables a more active and varied search for information. In addition, a direct exchange with other recipients can be supported (e.g., via e-mail, chat, forums) [24]. A total of 40.0% of the German population chooses the Internet as a source of information for health-related topics [24,25]. The study “Health Literacy Survey Germany 2 (HLS-GER 2)” (n = 2151) reported that 72.5% of the people aged between 46 and 64 and 40.3% of those aged 65 and older used Internet websites when searching for health-related topics [26]. The results of another study “Kommunikation und Information im Gesundheitswesen aus Sicht der Bevölkerung. Patientensicherheit und informierte Entscheidung” (Communication and information in the healthcare sector from the point of view of the population—Patient safety and informed decision-making; KomPaS) demonstrated that in particular, women younger than 65 years old use the Internet as a source of health information considerably more often than men. This trend starts to reverse among people aged 65 and over [23]. Although digital media provide users access to varied and evidence-based information material, in some instances the information on the Internet is difficult to understand or misinformation is presented [27]. An important requirement when dealing with information offerings is health literacy—the ability of individuals to be able to find, understand, critically appraise and use information, as well as to integrate it into their everyday lives [24]. 

As providers of health information, public health and health care stakeholders such as the SHI are obligated by the Social Security Code to educate and advise insured people (Social Security Code V, § 1) [28]. However, the SHI websites, which provide information for patients, exhibit qualitative deficiencies in some areas [27]. The information material from the health insurances is often poorly understood by the target group [29]. It is important that health information is prepared and presented adequately due to its influence on the decision of recipients for or against certain health behaviors such as the utilization of preventive services [24]. When dealing with health information, older people in particular face challenges [26,30,31] and they are reliant on adequate health information to make informed decisions for themselves and their families regarding their health [17]. 

As health information influences decision-making about (non-)utilization of preventive services [16,32], this study sought to provide an overview of web-based informational materials on colorectal cancer screening available from relevant public health and healthcare stakeholders in the federal state of Saxony-Anhalt, Germany and to evaluate the quality of information presented. 

## 2. Materials and Methods

### 2.1. Study Design

The study “Prävention im Alter Sachsen Anhalt—PrimA LSA” (Prevention in Old Age Saxony-Anhalt) was funded by the European Regional Development Fund and the federal state of Germany, Saxony-Anhalt (Project Nos: ZS/2019/07/99610, ZS/2020/06/145442) and a subproject in the research association “Autonomie im Alter—AiA” (Autonomy in old age) [33], which examines the utilization rates and the determinants of the utilization of preventive services by people aged 55 and older in Saxony-Anhalt, Germany. In this regard, relevant informational materials were systematically evaluated and scored on the basis of a comprehensive catalog of criteria, which was developed by the study team. The data collection occurred from 7 December 2020 to 15 July 2021. The study protocol of PrimA LSA was published elsewhere [34].

### 2.2. Development of the Evaluation Criteria

Various instruments for assessing the quality of health information already exist, both in English and German. These include, for example, the “Criteria for Judging the Quality of Patient Decision Aids” produced by the International Patient Decision Aid Standards (IPDAS) Collaboration [35,36], “DISCERN“—an instrument for judging the quality of written consumer health information on treatment choices [37], “Health On the Net—HONcode” for the ethical design of websites [38] and the criteria for evidence-based patient information [39]. However, it should be noted that the majority of the available instruments for the evaluation of health information were developed more than 10 years ago. The “Guideline Evidence-based Health Information” from the German Network for Evidence-based Medicine is a recently established recommendation for the production of high-quality health information material [14]. Due to the fact that the guideline goes beyond general quality criteria for information (e.g., target-group-oriented language style and accessibility), it was consulted for the development of the criteria catalog. In addition, the position paper “Good Practice Health Information”—also produced by the German Network for Evidence-based Medicine—was considered in the formulation of the evaluation of the quality of health information [18].

The catalog of criteria consisted of 7 main categories with 60 subcriteria (Table 1): (i) transparency (9 items), (ii) text design (10 items), (iii) content (13 items), (iv) language (5 items), (v) frequencies and statistical information (6 items), (vi) visualization (9 items) and (vii) accessibility (8 items). The study team discussed the first draft. Subsequently, pre-testing was conducted to verify the usability. Five members of the study team independently evaluated information material about vaccination against human papillomavirus. This vaccination was chosen as it is not a preventive service for the target population of the study. A test search was conducted for information material provided by four preselected public health stakeholders. The study members discussed inconsistencies and any identified weaknesses in the criteria catalog and made adjustments accordingly (e.g., specify the language used for the evaluation items).

### 2.3. Systematic Evaluation of Information Material

Relevant materials were web-based information offerings of SHI in Saxony-Anhalt (n = 13) as well as other relevant public health stakeholders at the federal or state level (e.g., associations, foundations, public bodies; n = 5). All materials were available online at the time of data collection. The selection of the actors occurred a priori on the basis of comprehensive prior research and discussions within the study team. 

### 2.4. Inclusion and Exclusion Criteria

To identify relevant informational materials, inclusion and exclusion criteria were determined. Sources that met the following criteria were included in the study:

(1) The materials were directed at persons with SHI and relevant healthcare stakeholders at a federal and state level.
(2) The materials could be found using the search field on the respective websites of the selected stakeholders.
(3) The informational materials were freely available when the search was conducted and either web-based or brochures were made available for download in PDF format.
(4) Information materials that were available in several formats (e.g., the same information text in the members’ magazine and on the website) were only included once in the analysis.

We excluded materials:

(1) that only described the clinical symptoms of colorectal cancer or the quality of clinical procedures such as the colonoscopy that did not focus on early detection of cancer in individuals without any symptoms,
(2) that were primarily directed at people aged under 55,
(3) that targeted other groups of people or institutions (e.g., healthcare providers, the press),
(4) that were predominantly based on a pictorial representation (e.g., posters),
(5) from websites that no longer existed, as indicated by error messages.

In addition, materials in a language other than German were excluded from the evaluation as it would have been necessary to consider them separately. The restriction of the language was motivated by the assumption that the majority of the eligible insured persons in the target population of those 55 years and older in Saxony-Anhalt were preferentially German-speaking [40].

### 2.5. Search Strategy

The search strategy was pre-defined and was binding for all websites. The Internet-based research took place from 7 December 2020 until 15 July 2021. It involved viewing various tabs and topic blocks on the web-sites of the public health stakeholders as well as looking at the first hits (maximum of 100) from searches using the search field on the respective websites. The search terms (in German) used for the topic of colorectal cancer screening included *bowel cancer*, *colonoscopy*, *colon cancer*, *rectal cancer* and *fecal immunological test* and its abbreviation *iFOBT* (English: FIT). Additionally, we also used more general terms such as *decision-making support*, *evidence-based information*, *early detection*, *early detection of cancer*, *cancer screening*, *prevention* and *screening*. 

### 2.6. Data Analysis

For the data analysis, a template was created and piloted to assess the identified information materials according to the criteria catalog. Two reviewers from the study team independently evaluated the materials. The question “Does it meet the criterion?” was answered using a 5-point Likert scale (“no” = 1, “more no than yes” = 2, “partially” = 3, “more yes than no” = 4, “yes” = 5). Each assessment was clarified and justified in writing by the reviewers in the template in an additional column for comments to increase the transparency of decisions made. Discrepancies between the reviewers were discussed and, if required, additional team members were consulted in order to reach a consensus. Further, the individual scores were added together. Taking into account criteria that could not be used due to missing data and that thus only received a score of “1”, a corrected maximum possible score was calculated and used to determine the relative maximum score. The evaluation scheme was adapted based on Wahl and Apfelbacher [41] and the systematic evaluation was accordingly modified in terms of the maximum possible score (Table 2). 

## 3. Results

**Figure 1:**
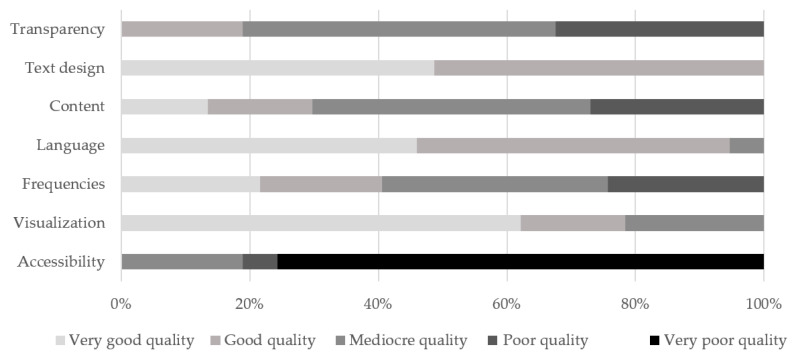
Quality of the evaluated materials (n = 37) on the basis of 7 main categories and 60 subcriteria in percent.

On the basis of the search strategy used, 1042 materials from 31 public health stakeholders were initially identified for screening. According to the pre-defined criteria, 184 materials were included in the analysis. After extraction of irrelevant informational materials, 37 materials in German from 16 stakeholders were evaluated. A total of 6 of 16 stakeholders delivered more than one topic-related information material (range: 1–11 materials per actor). In total, 27 websites and 10 PDF documents available online (e.g., brochures, flyers) were assessed. The informational materials included in the analysis were published between 2016 and 2020, and 14 materials did not include the publication date. The interrater reliability was calculated for 25% of the evaluated materials and revealed a 72% rater agreement, which, according to Landis and Koch [42], can be regarded as “good”. The evaluated materials as shown in Figure 1 were deemed to be of “mediocre quality” (median= 69%) overall. In terms of the criteria of “accessibility” (median = 31%) and “content” (median = 61%) the materials were rated “poor quality”. Three criteria achieved “good quality”: “language” (median = 88%), “visualization” (median = 88%) and “text design” (median = 90%).

**Figure 2:**
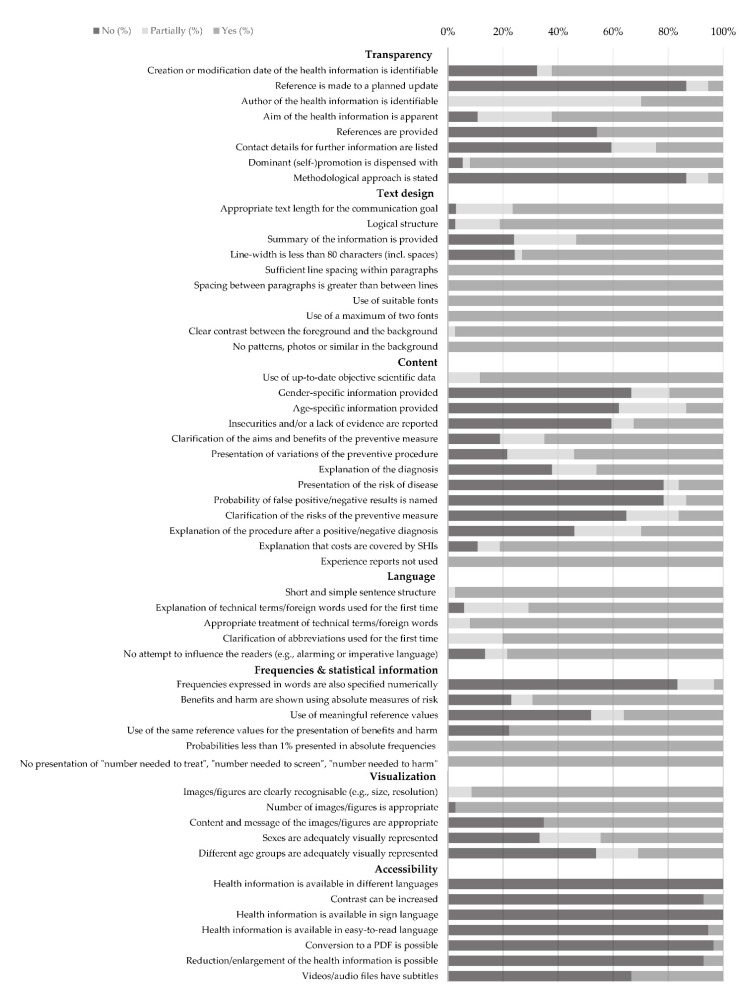
Results of evaluated health information on colorectal cancer screening (%).

Furthermore, the results of the entries (n= 6) in the respective search fields were in some instances user-unfriendly. For example, entering the search terms resulted in an excessively high number of hits (e.g., n ≥ 100 hits) with irrelevant content. In addition, search results were not specific to the topic, and the search function was inconvenient as all hits had to be manually viewed. In contrast, a search function was deemed user-friendly if the total number of hits was shown or if the search field was sensitive to spelling mistakes in the search term (e.g., colonoscopy). It was also judged to be user-friendly if suggestions appeared when entries were made in the search field (e.g., when the letter “D” was entered, suggestions like “Darm” (intestine), “Darmkrebs” (colorectal cancer/bowel cancer) and “Darmkrebsfrüherkennung” (colorectal cancer screening). A further issue identified was that the names of files containing informational materials (e.g., in PDF format) did not give an indication of the topic of the information. The results for the main categories presented below are covered in detail in Figure 2. 

### 3.1. Transparency

Of the nine items used to evaluate whether the presentation of the health information was transparent (Figure 2), the assessed quality varied between 30% and 88%. The overall score reflected a “mediocre quality” (median = 59%). The publication date could be identified for a majority of the materials (62%). However, only a minority made reference to a planned update of the informational materials (5%). The authors of the health information could only be identified for 11 of the materials, whereby 10 of those originated from the same actor. A concrete formulation of the information goal was present in 62% of the materials. More than half of the materials failed to include contact addresses for subsequent information searches (59%) and/or reference lists showing the sources used (54%). The majority of the materials (92%) did not engage in dominant advertising or self-promotion. Only two materials contained a methodological report detailing the creation of the health information. The subcriterion “existence of conflicts of interest/sponsors” was unable to be assessed for any of the materials, as this was not addressed by those issuing the information.

### 3.2. Text Design

The “text design” was evaluated on the basis of ten subcriteria (Figure 2). The scores ranged from 74% to 98%. An overall score of 90% indicated “good quality”. By and large, the materials satisfactorily met the subcriteria “appropriate text length” (70%), “logical structure” (81%) and “appropriate line width” (73%). Although 54% of the informational materials summarized the main points at the beginning or end of the text, concise information boxes were rather rare. All materials (n = 37) had sufficient line spacing, used suitable fonts with sufficient contrast between the text and the background and did not use distracting elements (e.g., patterns, photos) in the background. 

### 3.3. Content

Scores of the “content” (n = 13) yielded 32% and 100%. The criterion as a whole scored 61% (“mediocre quality”). Objective evidence-based data that was in line with the current state of knowledge (≤10 years) was used in 15 materials. However, the majority of the informational materials (n = 20) did not include a list of references. This meant that it was not possible to evaluate how up-to-date the data used in these materials were. Moreover, a majority neither provided a differentiated presentation of gender-specific (n = 24) nor age-specific (n = 23) aspects (e.g., potential risk factors, morbidity, mortality of colorectal cancer and the eligibility age for the screening). In case of one material, an evaluation of the item “gender-specific presentation” was not applicable as the information material specifically targeted insured men. Information detailing the risk of developing colorectal cancer was not contained in 78% of the materials. Moreover, possible uncertainties with regard to currently available scientific evidence were not explicitly or only partially mentioned in some materials (n = 25), and thus not clearly evident for users. Only five materials mentioned the probability of false negative or false positive test results. Only a few materials covered risks associated with the screening (e.g., side effects, misdiagnosis or overdiagnosis; n = 6). The “aims and benefits of the preventive measure” (65%) and “variations of the preventive procedure” (54%) were suitably presented in the majority of cases. The vast majority of the materials (81%) pointed out that SHI would cover the costs. All 35 materials either refrained from using narrative experience reports or made them clearly distinguishable from the factual information.

### 3.4. Language

The “language” was assessed to be “good quality” (median = 88%, range: 60–100%). Almost all materials (n = 36) employed a short and simple sentence structure. For the most, technical terms and foreign words were explained the first time they were used (n = 24). Frequently used technical terms were “polyp”, “intestinal polyp”, “colonoscopy”, “occult”, “adenoma” and “endoscopy”. Three materials did not contain any foreign words or technical terms, so the item “explanation of technical terms/foreign words when first used” could not be evaluated. Abbreviations were contained in 12 of 15 materials, which were also clarified when first used (e.g., “CT”, “iFOBT” (English: FIT), “G-BA”, “GKV” (English: SHI), “MRI”). Attempts to influence the readers by means of alarming, patronizing or imperative language were largely avoided (n = 32). Only five materials contained an appeal to the readers.

### 3.5. Visualization

The visual design was judged to be satisfactory with a score of 88% (“good quality”, range: 55–100%). The images used were evaluated based on the text with regard to whether they were an adequate representation of people’s age and gender. Only four materials provided appropriate depictions of different age groups. Eight materials adequately depicted people of different genders. However, it was not possible to evaluate 51% of the materials for this criterion, either because no images were used or because the gender of the people could not be clearly identified. Similarly, the appropriate depiction of people of different ages could not be evaluated for 65% of the materials as the age could not be identified. The recognizability (e.g., size and resolution of images) was judged to be appropriate for the majority of the materials (n = 21). There were 14 materials excluded from the evaluation, as they did not use any pictures, graphics or diagrams. None of the informational materials used diagrams. Thus, it was not possible to evaluate the subcriteria “reference values in the diagrams are presented logically”, “scale legends of the diagrams are appropriate, consistent and complete”, “diagram scale types are logical” and “diagrams have comprehensible legends”. Therefore, these criteria have not been included in Figure 2. 

### 3.6. Accessibility

The “accessibility” was scored as “very poor quality” (median = 29%, range: 20–60%). None of the evaluated materials were available in other languages. Only two materials enabled the contrast to be increased if required. Although 26 materials had a sign language option, this merely provided general information about the public health actor or the use of the website. The contents of the health information on colorectal cancer screening were not presented in sign language. A total of 27 materials provided an easy-to-read language option. However, the contents available did not go beyond details about the information provider or the use of the website. There was a total lack of information specifically about the preventive services in easy-to-read language. 

Two materials from one stakeholder had a text enlargement/reduction function. Three videos/ audio files contained subtitles. The remaining 34 materials could not be evaluated, as neither videos nor audio contributions were used. For a quarter of the materials, the text-to-speech function, the contrast settings, the possibility to enlarge/reduce the material, and the conversion to a PDF file were all inapplicable subcriteria as the materials were brochures in the form of PDF documents.

## 4. Discussion

To identify web-based informational materials about colorectal cancer screening and evaluate their quality, a systematic evaluation was conducted. The quality of health information plays an essential role pertaining to informed decision making for or against the utilization of preventive measures such as colorectal cancer screening [23]. The assessment covered information currently available from SHI with a dominant presence in the federal state of Saxony-Anhalt and from other public health and health care stakeholders at a federal and state level. The overall quality of the included 37 health informational materials was deemed to be “mediocre”. The results of the systematic evaluation demonstrated that the analyzed materials only partially comply with the National Cancer Plan’s call for comprehensible and target-group-oriented information on the benefits and risks of cancer screening [4] and the corresponding recommendations contained in the Guideline Evidence-based Health Information [14]. This was demonstrated by the inadequate presentation of the risks of colorectal cancer screening, the unsatisfactory accessibility of the information on the Internet and the commonly unstructured design of the stakeholders’ websites. In contrast, the majority of the materials used appropriate language and text design.

The risks associated with cancer screening were either mentioned very little in the evaluated health information or not at all. This finding is consistent with the results of other studies [19,43,44]. Günster et al. [43] pointed out that the existing information offerings on colorectal cancer screening are inadequate. In particular, they stated that information about the disadvantages of a colonoscopy (36%) was provided considerably less often than information about the benefits of the examination (75%) [43]. Dreier et al. [19] highlighted an imbalance between the presentation of the benefits and the risk. Tian et al. [44] identified also a lack of key information about risks and benefits by assessing web-based patient education materials in the United States. The results of the systematic evaluation support this claim.

In addition, statistical measures to aid comprehension (e.g., absolute or relative frequencies) were largely absent. These results are also consistent with the findings of Dreier et al. [19]. The analysis also found that the respective search field function was not user-friendly in some instances (e.g., a high number of imprecise hits). When there are many hits, one can assume that the users of the website will not look at all the search results. Adding a filter function might be helpful to overcome this issue, for example. 

With regard to the presentation of gender- and age-specific risks of developing the disease, the results show that the evaluated informational materials tend to portray colorectal cancer as a generally common form of cancer. In some cases, the materials also reported how many men or women are diagnosed with the disease each year. However, the risk of developing colorectal cancer with specific regard to gender and age is stated rarely, thereby making an individual risk assessment impossible. The KomPaS study [23] found that a search for health-related information is influenced by various determinants such as gender, age and socioeconomic status. Hence, information should be prepared specifically for different population groups, in order to enable individual contextualization of the information. 

Furthermore, the majority of the informational materials did not provide the necessary depth of information. Although Grewal et al. [45] evaluated English-language health information about colorectal cancer screening for quality, they reported insufficient information content for recipients to make informed decisions as well. With regard to our findings, one reason might be the structure of the websites. The information was in some cases unclearly and confusingly organized, which leads to a fragmentation of information. This manifested itself in multiple brief bits of information as well as numerous both internal and external links, which involved tediously clicking through pages. For example, within the website of one actor, eleven different topic-related materials were identified through cross-references. Hambrock [46] found that external links are seldom followed. This virtual transfer potentially risks users becoming disorientated, which can lead to a loss of interest. In addition, following the “bite, snack and meal” principle, it should also be possible for users to access further evidence-based information [47]. This approach enables users to gather information more quickly and to understand it, as information content is divided into three parts that are visually separated from each other: A catchy headline (“bite“) followed by a concise summary of the main points (“snack“) is supposed to arouse the interest of the users, so that they want to continue reading all the information (“meal“) [47]. 

Some public health stakeholders used external links to refer readers to other portals or to the policy holders’ brochure of the G-BA. It could be useful to present the information directly on the websites of the stakeholders, e.g., the SHI. In order to prevent readers being overwhelmed by the quantity of information, drop-down headings and tabs could be employed. Furthermore, if reference was made to the G-BA’s brochure for insured persons [9,10], it was often made to the outdated version from 2018. Although the only difference between the 2018 and 2020 versions of the information for insured persons was a changed visual design, reference should always be made to the current brochures. In addition, there were references not only to outdated informational materials, but also to online information that no longer existed. However, it was beneficial for users that several public health stakeholders additionally integrated health information from other institutions into their websites in order to draw attention to other valid sources of information regarding the early detection of colorectal cancer. 

In general, a positive trend is noticeable in the use of digital media. This increase has been simultaneously accompanied with an increase in the availability of information on the Internet [23,48]. Finding, understanding, appraising and applying health information—analog or from electronic sources such as the Internet—presupposes a certain degree of health literacy [22,24] and digital health literacy respectively [49,50]. However, previous studies found that almost three quarters of the general population face problems when assessing health information [26]. More than half of the German population has both a low level of health literacy (58.8%) and a low level of digital health literacy (75.8%), according to the HLS-GER 2 [13,51]. In particular, the health literacy level of people aged 65 and over is problematic (65.1%). Schaeffer et al. [51] stated that older age is associated with low digital health literacy. In order to strengthen the competence of the population in health-related contexts, the capabilities of individuals should be promoted and the contextual conditions should also be improved, e.g., by means of user-friendly communication of information by public authorities and SHI [29,52,53]. 

Furthermore, the results show that there is room for improvement in the design of web-based health information, especially information for population groups with auditive, visual and cognitive impairments. Health information should also be prepared specifically targeting people with impairments to enable them to make informed decisions. The UN Convention on the Rights of Persons with Disabilities recommends making information technologies available that simplify the access to (digital) information (e.g., audio material, use of easy-to-read language, videos with subtitles) [54]. At the health policy level, the Digital Healthcare Act (Digitales Versorgungsgesetz: DVG) is evidence of the increased significance of digital media in the context of healthcare and prevention [55]. With the help of the law, the access to digital information could also be improved for people with impairments as, among other things, the SHI covers the costs for listed Digital Health Applications by the Federal Institute for Drugs and Medical Devices, for example [55]. As digital media are firmly anchored in the everyday lives of the population as well as in different spheres of life (e.g., work, personal life), they can play a key role in the provision of information in the area of prevention and health promotion [23,48]. This could also be seen in the context of the COVID-19 pandemic. During the pandemic, the relevance of online digital information about infection control, prevention measures and the treatment of psychosocial problems steadily increased [56,57].

It can be stated that the provision of adequate health information on colorectal cancer screening promotes or strengthens informed decision-making about utilization of the service [58].

### Strengths & Limitations

This study relied on established and current guidelines and recommendations for the evaluation of health information [14,18]. In order to ensure the quality of the analysis and evaluation, it was conducted independently by at least two study team members. Furthermore, it should be considered that, in comparison with other instruments, the catalog of criteria developed goes beyond the correctness of the information and provides factors such as accessibility, text design and visualization. 

This study also has certain limitations. We excluded materials addressed to people aged under 55. The narrow focus on colorectal cancer information directed to the target group of people aged 55 and older was based on two considerations: On the one hand, the proportion of people with chronic diseases and multimorbidity increases due to higher life expectancy and advanced medical technologies [59]. On the other hand, the largest age group in East Germany are currently the birth cohorts from 1959 to 1968 due to the high birth rates in this period—they are the so-called “baby boomers” [60]. As the first baby boomers will retire from the workforce in 2025 and reach the age of 65 years, an increase in chronic diseases such as cancer can be expected in this birth cohort [61]. Legally recommended age-specific secondary preventive measures such as organized colorectal cancer screening starting at age 55 could prevent this [34].

The systematic evaluation did not include the users’ perspective. In order to achieve a comprehensive, target-group-oriented evaluation of informational materials, it is necessary to actively incorporate the point of view of the target population. This should be taken into account in future research approaches. Although pretesting of the catalog of criteria occurred, further aspects that could be improved were identified during the evaluation process. This will lead to an adjustment of the evaluation instrument. A revision of the instrument would need to be retested to a different research subject (e.g., mammography screening) to verify the validity and reliability in order to ensure the quality of the developed instrument. This might need to be carried out in a separate research project. With regard to the catalog of criteria, for instance, it should be noted that only a general evaluation of the correctness of the content was possible in line with the “Guideline Evidence-based Health Information” [36]. Moreover, the criteria catalog was developed for preventive services in general (e.g., cancer screening, vaccinations, medical and dental check-ups) and it can therefore be used in another context and for other age groups. In 2013, Dreier et al. [62] developed criteria to specifically determine the reliability of information in the context of colorectal cancer screening. It must be noted that Dreier et al. [62] had a narrow focus and mainly concentrated on evaluating the quality of the content. The catalog of criteria presented above goes beyond an assessment of the information content and considers other aspects to provide an overall picture of health information. The criteria such as design, transparency, language and accessibility of informational materials were included. However, for future evaluation of health information, the category “content” in the catalog should be expanded or adapted to include aspects for determining the correctness of the information and strength of evidence specifically for each preventive service being considered. The risk assessment of the preventive measure could be case-specific as well. 

In this context, a next step should be to develop a checklist for a guided creation of health information based on the catalog of criteria with the support of the study team, in order to assist public health and health care stakeholders in the conception of adequate and evidence-based health information. This checklist might provide stakeholders a summary of the most important aspects that (web-based) health information should contain.

## 5. Conclusions

The results of this systematic evaluation contribute towards identifying the existing weaknesses of currently available digital health information on colorectal cancer screening primarily from public health stakeholders of the federal state of Saxony-Anhalt, Germany. There is potential for improvement of the health information in terms of age-, gender- and target-group-specific presentation. The analysis of the materials revealed that the accessibility for users is unsatisfactory. Accessible web-based health information of an adequate quality should be available for all individuals. Thus, there is still a need for further development of health information, in particular for vulnerable populations.

## Figures and Tables

**Table 1:** Evaluation catalog of criteria on colorectal cancer screening information (modified from [14,18]).

	Main Categories & Subcriteria	No. of Items	Maximum Possible Points ^a^
Transparency	Creation or modification date of the health information is identifiable	9	45
	2. Reference is made to a planned update		
	3. Author of the health information is identifiable		
	4. Aim of the health information is apparent		
	5. References are provided		
	6. Contact details for further information are listed		
	7. Dominant advertising/self-promotion are dispensed with		
	8. Conflicts of interest/sponsors are named, if they exist		
	9. Methodological approach is stated		
Text design	10. Appropriate text length for the communication goal	10	50
	11. Logical structure		
	12. Summary of the information is provided		
	13. Line width is less than 80 characters (incl. spaces)		
	14. Sufficient line spacing within paragraphs		
	15. Spacing between paragraphs is greater than between lines		
	16. Use of suitable fonts		
	17. Use of a maximum of two fonts		
	18. Clear contrast between the foreground and background		
	19. No patterns, photos or similar in the background		
Content	20. Use of up-to-date objective scientific data	13	65
	21. Gender-specific information provided		
	22. Age-specific information provided		
	23. Insecurities and/or a lack of evidence are reported		
	24. Clarification of the aims and benefits of the preventive measure		
	25. Presentation of variations of the preventive procedure		
	26. Explanation of the diagnosis		
	27. Presentation of the risk of disease		
	28. Probability of false positive/negative results is stated		
	29. Clarification of the risks of the preventive measure		
	30. Explanation of the procedure after a positive/negative diagnosis		
	31. Explanation that costs are covered by the statutory health insurance (SHI)		
	32. Experience reports not used		
Language	33. Short and simple sentence structure	5	25
	34. Explanation of technical terms/foreign words used for the first time		
	35. Appropriate treatment of technical terms/foreign words		
	36. Clarification of abbreviations used for the first time		
	37. No attempt to influence the readers (e.g., by means of alarming/imperative language)		
Frequencies and statistical information	38. Frequencies expressed in words are also specified numerically	6	30
	39. Benefits and harm are shown using absolute measures of risk		
	40. Use of meaningful reference values		
	41. Use of the same reference values for the presentation of benefits and harm		
	42. Probabilities < 1% are presented in absolute frequencies		
	43. No presentation of “number needed to treat”, “number needed to screen” and “number needed to harm”		
Visualization	44. Images/figures are clearly recognizable (e.g., size, resolution)	9	45
	45. Number of images/figures is appropriate		
	46. Content and message of the images/figures are appropriate		
	47. Diagram scale types are logical		
	48. Diagrams have comprehensible legends		
	49. Scale legends of the diagrams are appropriate, consistent and complete		
	50. Reference values in the diagrams are presented logically		
	51. Sexes are adequately visually represented		
	52. Different age groups are adequately visually represented		
Accessibility	53. Health information is available in different languages	8	40
	54. Contrast can be increased		
	55. Health information is available in sign language		
	56. Health information is available in easy-to-read language		
	57. Conversion to a PDF is possible		
	58. Reduction/enlargement of the health information is possible		
	59. Videos/audio files have subtitles		
	60. Text-to-speech function is available		

^a^ Maximum total points: 300.

**Table 2:** Evaluation categories (modified from Wahl and Apfelbacher [41]).

Evaluation Categories	Score (Absolute)	Score (Relative, %)
Very good quality	300–272	>91
Good quality	271–212	90–71
Mediocre quality	211–152	70–51
Poor quality	151–93	50–31
Very poor quality	≤92	<31

## Data Availability

Not applicable.
